# Risk Factors Associated with Severe RSV Infection in Infants: What Is the Role of Viral Co-Infections?

**DOI:** 10.1128/spectrum.04368-22

**Published:** 2023-05-22

**Authors:** Kim Stobbelaar, Thomas C. Mangodt, Winke Van der Gucht, Lise Delhaise, Jasmine Andries, Valérie Gille, Cyril Barbezange, Annemieke Smet, Benedicte Y. De Winter, Jozef J. De Dooy, Tom Schepens, Els L. I. M. Duval, Paul Cos, Philippe G. Jorens, Stijn Verhulst, Peter L. Delputte

**Affiliations:** a Faculty of Medicine and Health Sciences, University of Antwerp, Antwerp, Belgium; b Laboratory of Microbiology, Parasitology and Hygiene, University of Antwerp, Antwerp, Belgium; c Laboratory of Experimental Medicine and Pediatrics, University of Antwerp, Antwerp, Belgium; d Department of Pediatrics, Antwerp University Hospital, Edegem, Belgium; e National Influenza Centre, Sciensano, Brussels, Belgium; f Infla-Med Centre of Excellence, University of Antwerp, Antwerp, Belgium; g Department of Gastroenterology and Hepatology, Antwerp University Hospital, Edegem, Belgium; h Department of Critical Care Medicine, Antwerp University Hospital, Edegem, Belgium; i Department of Intensive Care Medicine, Ghent University Hospital, Ghent, Belgium; University of Georgia College of Veterinary Medicine

**Keywords:** RSV, co-infection, risk factors, severity assessment, infectious disease, pediatric infectious disease, respiratory syncytial virus

## Abstract

The respiratory syncytial virus (RSV) represents the leading cause of viral lower respiratory tract infections (LRTI) in children worldwide and is associated with significant morbidity and mortality rates. The clinical picture of an RSV infection differs substantially between patients, and the role of viral co-infections is poorly investigated. During two consecutive winter seasons from October 2018 until February 2020, we prospectively included children up to 2 years old presenting with an acute LRTI, both ambulatory and hospitalized. We collected clinical data and tested nasopharyngeal secretions for a panel of 16 different respiratory viruses with multiplex RT-qPCR. Disease severity was assessed with traditional clinical parameters and scoring systems. A total of 120 patients were included, of which 91.7% were RSV positive; 42.5% of RSV-positive patients had a co-infection with at least one other respiratory virus. We found that patients suffering from a single RSV infection had higher pediatric intensive care unit (PICU) admission rates (OR = 5.9, 95% CI = 1.53 to 22.74), longer duration of hospitalization (IRR = 1.25, 95% CI = 1.03 to 1.52), and a higher Bronchiolitis Risk of Admission Score (BRAS) (IRR = 1.31, 95% CI = 1.02 to 1.70) compared to patients with RSV co-infections. No significant difference was found in saturation on admission, O_2_ need, or ReSViNET-score. In our cohort, patients with a single RSV infection had increased disease severity compared to patients with RSV co-infections. This suggests that the presence of viral co-infections might influence the course of RSV bronchiolitis, but heterogeneity and small sample size in our study prevents us from drawing strong conclusions.

**IMPORTANCE** RSV is worldwide the leading cause of serious airway infections. Up to 90% of children will be infected by the age of 2. RSV symptoms are mostly mild and typically mimic a common cold in older children and adolescents, but younger children can develop severe lower respiratory tract disease, and currently it is unclear why certain children develop severe disease while others do not. In this study, we found that children with a single RSV infection had a higher disease severity compared to patients with viral co-infections, suggesting that the presence of a viral co-infection could influence the course of an RSV bronchiolitis. As preventive and therapeutic options for RSV-associated disease are currently limited, this finding could potentially guide physicians to decide which patients might benefit from current or future treatment options early in the course of disease, and therefore, warrants further investigation.

## INTRODUCTION

Respiratory syncytial virus (RSV), recently reclassified to the species *Human Orthopneumovirus* (*hOPV*), is the leading viral cause of acute lower respiratory tract infections (LRTI) in young children worldwide (1). Up to 90% of children are infected with RSV at least once in the first 2 years of life. Clinical manifestations of RSV disease are usually mild in older children and adults, but primary infections are rarely asymptomatic, and severe LRTI such as bronchiolitis and pneumonia occur in up to 40% of infected children (2). It is estimated that, globally, RSV-LRTI are responsible for 3.2 million hospitalizations and 59,600 in-hospital deaths each year in children below 5 years of age alone (3). Despite the high medical, societal, and economic burden, both inside and outside hospital settings, treatment options for RSV remain elusive. Neither a safe and effective vaccine, nor antivirals, are currently available to control RSV infections, except for the monoclonal antibody palivizumab (Synagis), which is reserved exclusively for high-risk infants, such as premature infants, or children with cardiopulmonary disease, mainly because of the high cost and need for monthly injections (4).

Currently, it is unclear why some RSV-infected children become severely ill, while others have only mild symptoms. This divergent disease severity can be explained at least partially by the complex and multifactorial pathogenesis of the virus, in which direct virus-induced cytotoxicity, virus-induced immunopathology, host genetic constitution, and environmental factors each play a crucial role (5). Although historically, research focused primarily on host immune responses as an explanation for the wide array of clinical manifestations, it is now becoming increasingly evident that viral characteristics might also be implicated (6, 7). Previous research in our own group therefore focused on several of these virus-related factors and further added to this presumption (8).

The role of interactions between RSV and secondary pathogens in disease severity, however, remains questionable. Bacterial superinfections during an RSV bronchiolitis have already been established as a risk factor for a more severe clinical picture (9, 10). In the last years, viral co-infections have gained more attention as well, partly due to the increased and more widespread use of molecular methods such as multiple-agent polymerase chain reactions (PCR’s) (11). Rates of viral co-infection in RSV bronchiolitis range from 5% to 35% (12–14), and Rhinovirus (RV) is the most commonly detected agent (15–17). A recent systematic review regarding the severity of RSV single versus multiple viral co-infections could not find an association between the presence of RSV co-infections and disease severity, except for RSV-human metapneumovirus (hMPV) co-infections, but this study lacked sufficient power to reach firm conclusions (18).

Therefore, our study will investigate the impact of viral co-infections on several disease severity indices, such as the need for intensive care, supplementary oxygen, the length of hospital stay, and bronchiolitis severity scores, in patients suffering from RSV positive LRTI in the winter seasons of 2018 to 2020 at a tertiary hospital.

## RESULTS

### Demographic and clinical characteristics of patients.

**FIG 1:**
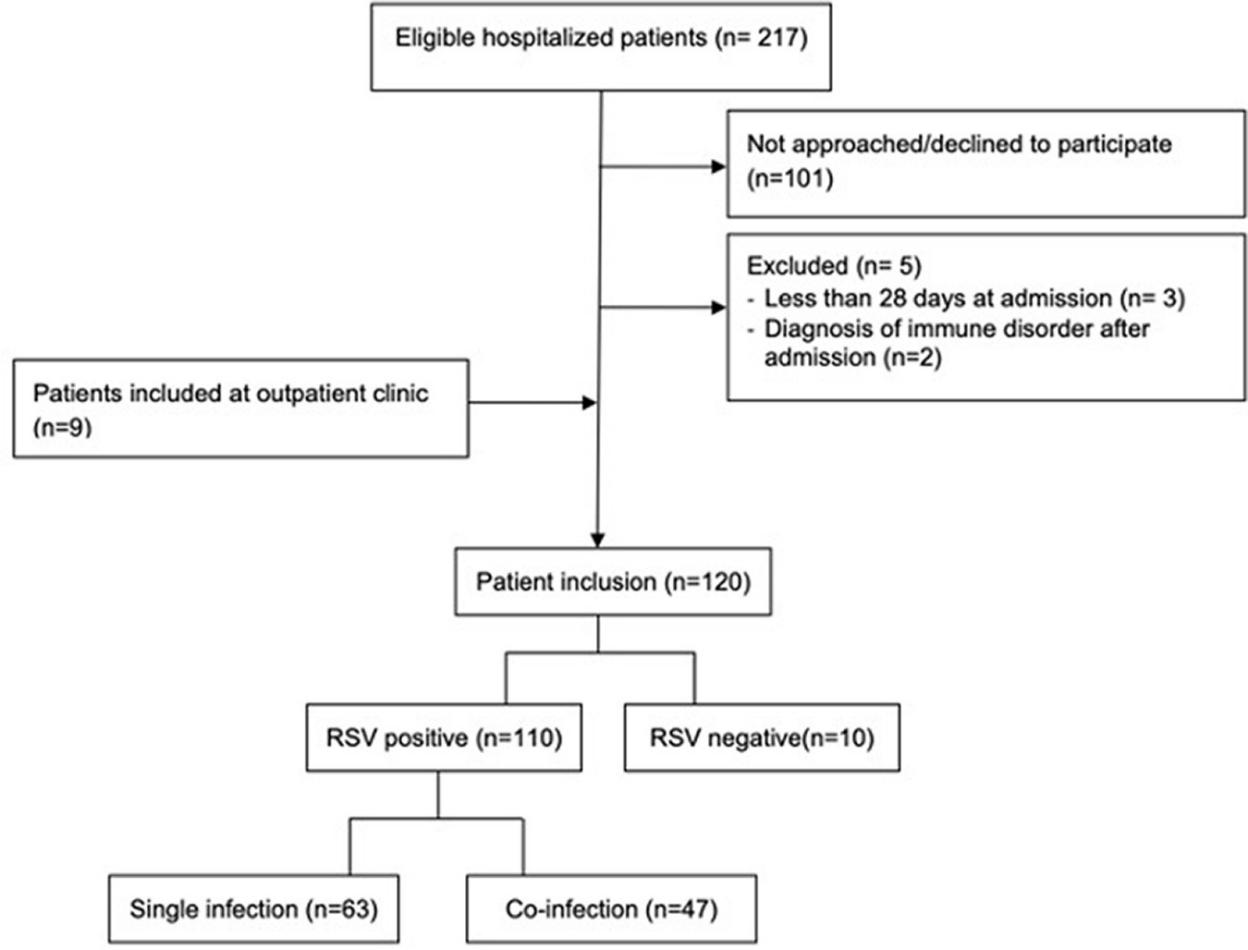
Patient inclusion flow chart.

During the study period between 2018 and 2020, 120 patients aged 28 days up to 2 years old presenting with clinical signs of an acute lower respiratory tract infection at the Antwerp University Hospital were enrolled in the study (Fig. 1). Of the 120 included patients, 110 had a positive PCR test for RSV (91.7%). General and clinical characteristics of these RSV positive patients are summarized in Table 1. The median age of RSV positive patients in our study was 5.0 months old (2 to 9.9) and there was a slight male predominance (62%). Most of the included patients were admitted to the general pediatric ward (74%), with a median length of stay of 4 (2 to 6) days, 18.2% necessitated ICU admission and 8.2% were ambulatory patients. More than half of the hospitalized patients needed supplementary oxygen administration, with a median duration of oxygen requirement of 3 (1.5 to 5) days.

**TABLE 1:** Patient characteristics

Study population	RSV		RSV-A		RSV-B		
		*N* (%)		*N* (%)		*N* (%)	
	*n*	110	*n*	41^*a*^	*n*	68^*a*^	*P*
General characteristics^*c*^							
Male sex (*n*, %)	68	110 (61.8)	22	41 (53.7)	46	68 (67.6)	0.16
Prematurity (*n*, %)	11	110 (10)	3	41 (7.3)	8	68 (11.8)	0.53
Small for gestational age (*n*, %)	10	109^*b*^ (9)	2	41 (4.8)	7	67^*b*^ (10.4)	0.48
Daycare attendance (*n*, %)	56	105^*b*^ (53.3)	20	38^*b*^ (52.6)	36	66^*b*^ (54.5)	1
Breast feeding (*n*, %)	65	110 (59)	23	41 (56)	42	68 (61.8)	0.69
Parental atopy (*n*, %)	18	105^*b*^ (17.1)	4	39^*b*^ (10.3)	14	65^*b*^ (21.5)	0.18
Parental smoking (*n*, %)	29	103^*b*^ (28.2)	8	38^*b*^ (21.0)	20	64^*b*^ (31.3)	0.36
Age (mo; median, Q1 to Q3)	5.0	2 to 9.9	5.1	1.8 to 10.5	5.0	2.3 to 10.1	0.62
Clinical parameters							
Dyspnea (*n*, %)	82	110 (74.5)	27	41 (65.9)	54	68 (79.4)	0.17
Anorexia (*n*, %)	89	110 (80.9)	33	41 (80.5)	55	68 (80.9)	0.96
Fever (*n*, %)	90	110 (81.8)	34	41 (82.9)	55	68 (80.9)	0.79
Cyanosis (*n*, %)	5	110 (4.5)	0	41 (0.0)	5	68 (7.4)	0.16
Tachypnea on admission (*n*, %)	73	110 (66.4)	22	41 (53.7)	48	68 (70.6)	0.07
Tachycardia on admission (*n*, %)	33	110 (30)	10	41 (24.4)	23	68 (33.8)	0.34
Primary outcome measures^*e*^							
PICU admission (*n*, %)	20	110 (18.2)	11	41 (26.8)	9	68 (13.2)	0.12
Duration of hospitalization (days; median, Q1 to Q3)	4	2 to 6	4	2 to 7	4	2 to 5	0.56
Desaturation on admission (*n*,%)	25	110 (22.7)	7	41 (17.1)	18	68 (26.5)	0.26
Oxygen administration (*n*,%)	65	110 (59.1)	21	41 (51.2)	44	68 (64.7)	0.23
BRAS (median, Q1 to Q3)	2	2 to 3	2	2 to 3	3	2 to 4	0.09
Secondary outcome measures							
Duration of O_2_ administration (days, median, Q1 to Q3)	3	1.5 to 5	3	1.5 to 5	2.25	1.1 to 5	0.89
Maximum O_2_ flow (l/min, median, Q1 to Q3)	8	1 to 13.5	10	1 to 14.5	1.25	1 to 12	0.13
Maximum F_i_O_2_ (%; median, Q1 to Q3)	26	24 to 40	38	24 to 55	24	24 to 30	**0.02** ^*d*^
Intubation (*n*, %)	3	110 (2.7)	0	41 (0.0)	3	68 (4.4)	0.29
ReSViNET score (median, Q1 to Q3)	8	5 to 11.25	7	3 to 11	9	6 to 12	0.12

a One patient tested positive for both RSV-A and RSV-B.

b Data is lacking for some patients.

c Statistical analysis was performed using Pearson's chi-squared, Fisher’s exact, or Mann-Whitney-U test, as appropriate.

d Significant *P*-values in bold.

e PICU, pediatric intensive care unit; BRAS, Bronchiolitis Risk of Admission Score; F_i_O_2_, fraction of inspired oxygen.

In total, 41 patients had an RSV-A infection, 68 patients had an RSV-B infection, and one patient had a dual infection with RSV-A and B. Comparison between patients of these two subtypes showed no remarkable differences, except for a statistically significant higher F_i_O_2_ in the RSV-A group (Table 1).

**FIG 2:**
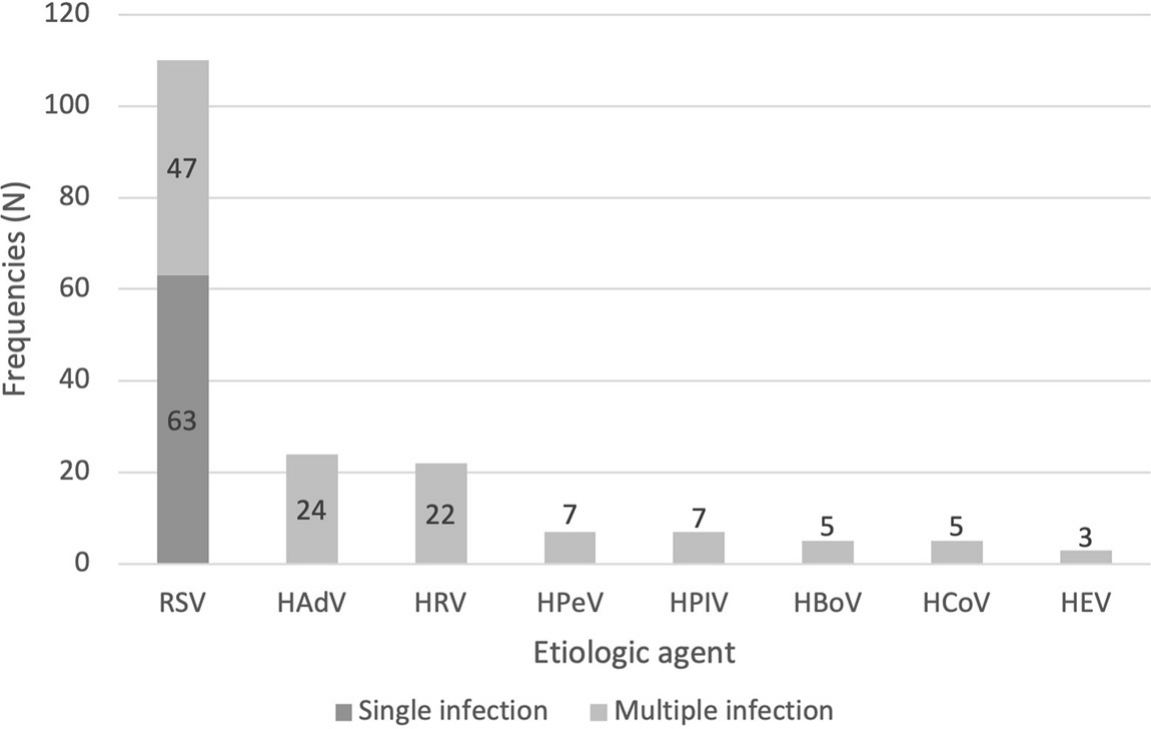
Distribution of etiologic agents in RSV positive samples collected in winter seasons of 2018 to 2019 and 2019 to 2020 (*N* = 110).

A multiplex RT-qPCR showed that in 42.7% of the samples (47/110), multiple viral agents were present. Of these, 29, nine, five, and three samples had co-infections with, respectively, two, three, four, or five different viruses. One sample tested negative for all viruses that are screened for with the multiplex RT-qPCR respiratory panel. The most prevalent species, besides RSV, were HAdV (21.8%), HRV (20.0%), HPeV (6.4%), HPIV (6.4%), HBoV (4.5%), HCoV (4.5%), and HEV (2.7%) (Fig. 2).

### Impact of viral co-infections.

Table 2 depicts the comparison of general characteristics, clinical parameters and severity surrogates between patients suffering from RSV single infections and patients with RSV co-infections. Looking at severity indices, we observed a significantly higher number of pediatric intensive care unit (PICU) admissions (*P* = 0.01), longer LOS (*P* = 0.02), and a higher disease severity score on the Bronchiolitis Risk of Admission Score (BRAS) (*P* = 0.01) in children with a single RSV infection. Furthermore, RSV co-infections were more prevalent in boys (*P* = 0.01) and patients in this group were on average older than patients in the single infection group (*P* = 0.03). Moreover, daycare attendance (*P* < 0.001), parental atopy (*P* = 0.02), and parental smoking (*P* = 0.04) were also more common in the co-infection group. Clinical parameters didn’t differ significantly between the two groups.

**TABLE 2:** RSV single versus co-infections

Population	Single RSV infection		RSV co-infection		
		*N* (%)		*N* (%)	
	*n*	63	*n*	47	*P*
General characteristics^*b*^					
Male sex (*n*, %)	32	63 (50.8)	36	47 (76.6)	**0.01** ^*c*^
Prematurity (*n*, %)	8	63 (12.7)	3	47 (6.4)	0.35
Small for gestational age (*n*, %)	6	62^*a*^ (9.7)	4	47 (8.5)	1
Daycare attendance (*n*, %)	24	62^*a*^ (38.7)	32	43^*a*^ (74.4)	**<0.001**
Breast feeding (*n*, %)	40	63 (63.5)	25	47 (53.2)	0.28
Parental atopy (*n*, %)	6	61^*a*^ (9.8)	12	44^*a*^ (27.3)	**0.02**
Parental smoking (*n*, %)	12	59^*a*^ (20.3)	17	44^*a*^ (38.6)	**0.04**
Age	3.3	1.6-8.8	5.8	3.9 to 11.2	**0.03**
Clinical parameters					
Dyspnea (*n*, %)	43	63 (68.3)	39	47 (83)	0.08
Anorexia (*n*, %)	51	63 (81)	38	47 (80.9)	0.99
Fever (*n*, %)	50	63 (79.4)	40	47 (85.1)	0.44
Cyanosis (*n*, %)	3	63 (4.8)	2	47 (4.3)	1
Tachypnea on admission (*n*, %)	42	63 (66.7)	28	47 (59.6)	0.44
Tachycardia on admission (*n*, %)	20	63 (32.3)	13	47 (27.7)	0.61
Primary outcome measures^*d*^					
PICU admission (*n*, %)	17	63 (27)	3	47 (6.4)	**0.01**
Duration of hospitalization (days, median, Q1 to Q3)	4	3 to 7	3	2 to 5	**0.02**
Desaturation on admission (*n*, %)	17	63 (27)	8	47 (17)	0.22
O_2_ administration (*n*, %)	41	63 (65.1)	24	47 (51.1)	0.14
BRAS (median, Q1 to Q3)	3	2 to 4	2	1 to 3	**0.01**
Secondary outcome measures					
Duration of O_2_ administration (days; median, Q1 to Q3)	3	1.5 to 5	2	1 to 4.875	0.49
Maximum O_2_ flow (l/min; median, Q1 to Q3)	8	1 to 12.5	1.5	1 to 14	0.73
Maximum F_i_O_2_ (%, median Q1 to Q3)	28	24 to 47	24	24 to 30	0.16
Intubation (*n*, %)	3	63 (4.8)	0	47 (0)	0.26
ReSVinet score (median, Q1 to Q3)	9	5 to 12	8	5 to 11	0.43

a Data are lacking for some patients.

b Statistical analysis was performed using Pearson's chi-squared, Fisher’s exact, or Mann-Whitney-U test, as appropriate.

c Significant *P*-values in bold.

d PICU, pediatric intensive care unit; BRAS, Bronchiolitis Risk of Admission Score; F_i_O_2_, fraction of inspired oxygen; .

We compared RSV single infections (*n* = 63) with the two most prevalent dual infections, RSV-ADV co-infection (*n* = 13) and RSV-RV co-infection (*n* = 10), because all other dual co-infections were low in frequency (*n* < 3). The single RSV infections were associated with higher BRAS scores, compared to RSV-ADV and RSV-RV co-infection (*P* = 0.047 and 0.03, respectively), but there were no other statistically significant differences in severity indices.

As young age, male sex, and prematurity are all known risk factors for serious RSV disease, and thus potential confounders, a logistic regression was performed to ascertain the effects of these risk factors on the likelihood of PICU admission. This analysis showed that patients with a single RSV infection had a 5.9 times higher risk of PICU admission and none of the other variables were of significance (Table 3). Likewise, a Poisson regression was run to evaluate the influence of these risk factors on the duration of hospitalization (Table 4) and the BRAS score (Table 5) and showed that patients with a single RSV infection had a 25% longer length of hospital stay and 31% higher BRAS-score. An interaction term consisting of the variables “age” and “single versus co-infections” was tested, but was statistically nonsignificant in all three regression analyses and was, therefore, removed out of the regression equations. Although not statistically significant, the number of premature infants was higher in the single infection group. Prematurity was therefore included as covariate and showed to be of significant influence on the length of hospital stay only. An interaction term was subsequently tested, but was nonsignificant and thus removed out of the analysis.

**TABLE 3:** Logistic regression with PICU admission as response variable^*a*^

Variable	B	SE	Wald	*df*	Sig.	Exp(B)	Lower 95% CI	Upper 95% CI
Single vs co-infection	1.775	.688	6.657	1	**.010**	5.903	1.532	22.742
Male sex	.586	.557	1.106	1	.293	1.797	.603	5.357
Prematurity <37 weeks	.277	.773	.129	1	.720	1.319	.290	5.999
Age (months)	−.055	.053	1.059	1	.303	.947	.853	1.051
Constant	−2.822	.825	11.689	1	<.001	.059		

a Significant *P*-values in bold.

**TABLE 4:** Poisson regression with length of hospital stay as response variable^*a*^

Parameter	B	SE	Wald	*df*	Sig.	Exp(B)	Lower CI	Upper CI
(Intercept)	2.053	0.1403	214.081	1	0.000	7.792	5.918	10.258
Single vs co-infection	0.223	0.0971	5.285	1	**0.022**	1.250	1.033	1.512
Age (mo)	−0.027	0.0089	9.305	1	**0.002**	0.973	0.956	0.990
Male sex	0.114	0.0961	1.414	1	0.234	1.121	0.929	1.353
Prematurity <37 wks	0.500	0.1208	17.105	1	<**0.001**	1.648	1.301	2.089

a Significant *P*-values in bold.

**TABLE 5:** Poisson regression with BRAS score as response variable^*a*^

Parameter	B	SE	Wald	*df*	Sig.	Exp(B)	Lower CI	Upper CI
(Intercept)	0.784	0.2344	40.743	1	<0.001	2.190	1.383	3.466
Single vs co-infection	0.273	0.1309	4.336	1	**0.037**	1.313	1.016	1.697
Age (mo)	−0.033	0.0121	7.568	1	**0.006**	0.967	0.945	0.990
Male sex	−0.028	0.1257	0.049	1	0.824	0.972	0.760	1.244
Prematurity <37 wks	−0.220	0.2116	1.076	1	0.299	0.803	0.530	1.216

a Significant *P*-values in bold.

Although both tested clinical scoring systems showed the same effect, with higher scores in the single RSV infection group, this difference was only statistically significant for the BRAS score. As age is a criterion in the BRAS score and not in the ReSViNET score, this might have played a role. We have therefore re-analyzed the BRAS score, without age as criterion and this showed that the BRAS score still differed significantly between the two groups, with a median score of 3 (IQR 2 to 3) in the single infection group and 2 (IQR 1 to 3) in the co-infections group (*P* = 0.017).

## DISCUSSION

RSV is a major cause of hospitalization, especially in young children, and is therefore considered to be a global health priority. RSV is responsible for a broad spectrum of clinical manifestations and it is still not completely understood why some children become severely ill, while others display only mild symptoms. As the role of viral co-infections in RSV pathogenesis is still largely unknown, we wanted to evaluate the effect of viral co-infections on disease severity in a series of patients with an RSV positive lower respiratory tract infection. Our findings suggest that young children suffering from a single RSV infection have a higher risk of PICU admission, longer length of hospital stay, and are also more severely ill, as quantified by the BRAS score, compared to patients suffering from an RSV co-infection. Even more surprising, there were significantly less males, children attending daycare, and children of atopic and/or smoking parents, which are all known risk factors for severe RSV disease, in this single RSV infection group with overall higher RSV disease severity.

Children in this single infection group were significantly younger compared to patients suffering from an RSV co-infection. As age could be of potential influence, regression analysis was run to assess the contribution of different known risk factors to the possibility of PICU admission, the length of hospital stay, and the BRAS score. This analysis showed that, although age was of significant influence on the length of hospital admission and the BRAS score, this was not the case for the probability of PICU admission. Furthermore, the interaction term did not contribute in a meaningful way to the predictive ability of the regression equations, suggesting that age did not affect the influence the presence or absence of a co-infection has on the disease severity in RSV infected patients.

Literature about the impact of RSV co-infections on disease severity is increasing due to the widespread availability of PCR assays that detect multiple pathogens, but this topic remains controversial. Li et al. recently published a systematic review and came to the conclusion that there is no association between co-infections with RSV and disease severity, except for RSV-hMPV co-infections, which were associated with higher risk of PICU admission (18). In this study, however, we did observe a significantly lower disease severity in RSV co-infections.

Why single RSV infections in our study result in more severe disease compared to co-infections is not entirely clear. First, when multiple viruses are detected by molecular tests, the question arises whether all detected viruses are indeed causative for the clinical picture, or whether they are simple innocent bystanders. They could also represent successive infections, of whom a persistent genome is detected in the absence of viral replication (19). However, although viral respiratory infection can present asymptomatically and hCoV and hBoV are indeed frequently detected in healthy controls, RSV and hMPV rarely do. Furthermore, as we do not ask the patients for blood samples, we have no serology data for these patients and we cannot distinguish between primary or secondary RSV infections. However, upon inclusion, every parent was asked whether this is the first RSV infection, and this was the case for all patients. Although this does not exclude that some children might have had an earlier RSV infection that was not diagnosed as such, given that there was only 2,5 months median difference between both groups, we do not expect major differences for possible previous RSV infections between the two groups. Another possibility is that a prior or simultaneous infection with another virus stimulates the host’s antiviral immune response which reduces the capacity of RSV to replicate to high levels, thereby reducing RSV’s capacity to induce disease (20, 21). Moreover, other researchers have postulated that in co-infections with RSV, the severity is mainly determined by the RSV infection rather than by the coinfecting virus (14). A hypothesis as to why single infections are associated with more severe disease is that the skewed immune response that RSV, unlike other respiratory viruses, induces, is prevented by co-infection with another virus. Nicholson et al. indeed described that the clinical picture of bronchiolitis is determined by a virus-specific response, rather than a generalized inflammatory response to all viral pathogens (22). Therefore, RSV-specific immune response and immunopathology could be counteracted and even neutralized in case of a simultaneous infection with multiple viruses. Serum levels of TIMP-1 and PDGF-BB have been implicated in this hypothesis (23). Interestingly, in a recent Spanish paper, patients with RV/EV + multiple viral codetections in whom RSV was also detected showed a different clinical picture in comparison to patients with a dual RV/EV + RSV codetection, having a significantly shorter PICU stay and lower need for mechanical ventilation (24). They hypothesized that different patterns of viral infection (single infection versus dual or multiple co-infections) may lead to different changes in respiratory microbial communities and different grades of airway inflammation (23). It has also been suggested that changes induced by RSV in nasopharyngeal bacterial microbiome could cause a more severe LRTI, by modulating the host immune response (25). Therefore, one could hypothesize that the difference in disease severity observed between single and co-infections in our study could at least partially be explained by a respiratory microbiota imbalance.

The finding that children with co-infections are older compared to children with a single RSV infection is not completely surprising and was also reported by Rotzén-Östlund et al. (26). The main hypothesis for this is that this might be due to more frequent contact with other children in various settings, including the parent's social activities and daycare attendance, which tends to start after the age of 3 months (26). Our findings indeed show that almost 75% of patients in the co-infections group attended daycare.

In our study, we found that parents of children with RSV co-infections were more atopic and there was also significantly more exposure to smoking in these patients. Other authors also investigated the relation of RSV co-infections with a family history of atopy or smoke exposure, but were unable to demonstrate a significant difference between RSV single and co-infections (15, 16, 27, 28). One study, however, recently demonstrated a higher risk of sensitization to aeroallergens at 3 years after the initial bronchiolitis in case of RSV-RV co-infection (29).

We were unable to identify a specific difference in clinical presentation between RSV single and co-infections, which is in accordance with other authors (27, 30, 31). Fever has been reported to be more frequent (32) and higher in peak temperature (33) in those with RSV co-infection, while we, just as Sung et al. (in RSV-hMPV co-infections) (34), did not notice such a difference.

No difference was seen in any of the severity surrogates between RSV-A and RSV-B positive patients, with the only exception of a higher maximal F_i_O_2_. An increased disease severity has previously been allocated to RSV-A (35–37) or RSV-B (38, 39) disease. Our data, however, indicate that the RSV type had no relation to RSV severity, and as such, agrees with many other authors (40, 41). Controversy on this topic can be at least partially explained by heterogeneity in study design, such as differences in outcome parameters, study populations, and duration of symptoms at the time of study enrollment and sampling (40).

The world is currently still fighting the COVID-19 pandemic. Few epidemiological studies have been published so far describing co-infections with RSV in children with documented COVID-19 infection (42–44). This paucity of literature regarding RSV co-infections in children with COVID-19 can be at least partially explained by the initial worldwide dramatic decline in RSV incidence (45). The incidence of viral co-infections in pediatric COVID-19 patients ranged from 5% to 7% (42, 43, 46). A recent systematic review investigating the role of respiratory co-infection with influenza or RSV in the clinical severity of COVID-19 patients could not find a correlation between RSV-COVID-19-co-infections and death among COVID-19 patients, the only outcome for which they had sufficient data to allow for analysis (47). Likewise, a South African COVID-19 study could not demonstrate a clear difference in disease severity between single and co-infections (44). With the resurgence of RSV epidemics, whether co-infection of SARS-CoV-2 with other viruses such as RSV aggravates bronchiolitis severity will be a most interesting study topic in the forthcoming RSV endemic seasons.

Our study has a few limitations. The main limitation is the small sample size, especially of certain subgroups. For example, the assessment of patient care as outcome measure is complicated by the small number of outpatients that have been included. On the other hand, as the decision to admit a young patient to the hospital is mainly based on the sometimes subjective evaluation of the primary physician, this could form a bias and might render type of patient care less suitable as severity index. Additionally, although patients were included prospectively and samples were taken as early as possible, the evaluation of the patient files was done retrospectively, thus sometimes resulting in gaps in our data collection or misinterpretation of the physician’s evaluations. Lastly, the lack of a sufficiently validated gold standard scoring system for disease severity in patients suffering from bronchiolitis complicates adequate assessment and comparison of patients. Numerous clinical scoring systems for the assessment of bronchiolitis are currently available and extensive work has been taken to assess the characteristics and validation process of these instruments. The ReSVinet group published their scoring system in 2016, thereby contributing to the field with a better validated tool, as was demonstrated by their elaborate comparison (48–50). Although data assessing the reliability are still lacking, the ReSVinet score had by far the highest number of positively rated validation criteria (49, 50). After a thorough literature review, we therefore chose to use the ReSVinet score for this research and compared it with the BRAS score, as this instrument was deemed the best by the systematic review conducted by Rodríguez-Martínez et al. (48, 51, 52). In this study, both scoring systems showed a different outcome, with a significant effect observed using the BRAS, but not with the ReSViNET scoring system. This emphasizes the need of a properly validated and universally applicable scoring instrument.

In conclusion, in our study, patients with a single RSV infection had increased disease severity compared to patients with RSV co-infections, as indicated by higher rates of PICU admission, longer length of stay, and higher BRAS scores, irrespective of the age of the patients. This could suggest that the absence or presence of viral co-infections might influence the course of RSV bronchiolitis. However, our study had a heterogeneous study population and small sample size, warranting more large-scale studies to confirm these findings. Furthermore, such studies should include a comparison of both the BRAS and ResVinet scoring system, as this study showed that while significant differences were observed using BRAS, this was not the case with the ResVinet scoring system.

## MATERIALS AND METHODS

### Patient inclusion and clinical sample collection.

Previously healthy children aged 28 days (i.e., neonates, according to the WHO definition) up to 2 years old presenting with clinical signs of an acute LRTI were included prospectively during an outpatient visit or hospitalization at the general pediatric ward or PICU of the Antwerp University Hospital (Edegem, Belgium). Since RSV is the main etiologic agent of interest for this study, patients were only eligible for inclusion if they had a positive or unknown diagnostic PCR result for RSV. Patients with a known negative PCR result for RSV beforehand were therefore not approached. We aimed to include patients with an RSV-induced LRTI, such as bronchiolitis and pneumonia, but variability exists in the definitions for these diseases. Patients were therefore included if they had at least one specific lower respiratory tract sign reported by the child’s caregiver or the responsible physician, such as tachypnea, dyspnea, and increased work of breathing; and/or abnormal auscultatory findings, such as rhonchi, crepitations, wheezing, or bronchial breath sounds. Inclusion took place during the two consecutive winter seasons of 2018 to 2019 and 2019 to 2020, respectively, from week 42 until week 8 of the next calendar year. All patients were included before the COVID-19 pandemic. Patients with underlying, symptomatic cardiopulmonary disease or immunodeficiency were excluded. Written parental informed consent was registered before inclusion. The Ethical Committee of the Antwerp University Hospital approved this study (nr. 16/46/491).

After inclusion, parents completed a questionnaire on demographic and clinical data. Afterwards, nasal secretions were collected via nasopharyngeal aspiration via a standardized procedure: 2mL of physiologic saline solution were injected in one of both external nostrils and this solution was aspirated through a flexible rubber hose in a closed suction system. Immediately after aspiration, the samples were preserved at 4°C and transferred to the lab for further processing.

### RNA extraction and multiplex qPCR.

Within 1 h after arrival at the lab, every nasal sample was resuspended in Hanks Balanced Salt Solution (HBSS) containing 20% sucrose, aliquoted, snap-frozen, and stored at −80°C until further processing. Subsequently, after thawing, RNA was extracted using the QIAamp viral RNA minikit (Qiagen, Hilden, Germany), according to the manufacturer’s recommendations and RNA concentration and quality were determined with the NanoDrop Spectrophotometer. RNA with an OD 260/280 ratio greater than 1.8 was considered to be of good quality. Next, a multiplex quantitative reverse transcription-PCR (RT-qPCR) was performed using the SuperScript III Platinum One-Step RT-qPCR kit (Thermo Fisher Scientific, Inc.) with specific oligonucleotide primers for RSV-A and RSV-B as well as 14 other common respiratory viruses, including human parainfluenza viruses (hPIV), human rhinoviruses (HRV), human metapneumoviruses (hMPV), human parechoviruses (hPeV), human bocaviruses (hBoV), human coronaviruses (hCoV), human adenoviruses (hAdV), and human enteroviruses (hEV). Testing for influenza was not done, as the RSV and influenza seasons at that time did not overlap in our region and over 75% of children included in this study already had a diagnostic nasal sampling for RSV and influenza A/B performed preinclusion, which was negative for influenza for all of them. The RT-qPCR was run on the MX 3005 Instrument by Stratagene and Cq-values below 40 were counted as positive.

### Outcome measures for severity assessment.

As validated gold standards for the assessment of disease severity in young children suffering from bronchiolitis are lacking, we used the following parameters as primary outcome measures: need for PICU admission, duration of hospitalization, saturation (SaO_2_) on admission, and need for supplementary oxygen administration. As secondary outcome parameters, we also included duration of oxygen administration, maximum administered fraction of inspired oxygen (F_i_O_2_), maximum oxygen flow, and need for intubation. Furthermore, two different severity scoring systems were included. The first one is the BRAS by Marlais et al. (51), as this instrument was determined the best currently available by a recent systematic review (53). This scoring system was used as primary outcome. Because the ReSVinet scoring system developed by Justicia-Grande et al. (48) was not included in this systematic review, we also included this scoring system as secondary, exploratory outcome measure, as it seems to be one of the most validated assessment tools currently available (49). The scores were computed retrospectively for each patient by two independent investigators, based on the first clinical assessment available in the electronic patient file. In case of score discrepancy, differences were discussed, and a final score allocated.

### Statistical analysis.

Continuous or categorical variables with nonnormal distributions are expressed as medians (Q1 to Q3), dichotomous categorical variables are presented as frequencies (%). Bivariate analyses, including Pearson’s chi-squared test, Fisher’s Exact test, and Mann-Whitney U test, were performed to compare group proportions and/or distributions, as appropriate. When suitable, odds ratios (OR) or incident rate ratio’s (IRR) were computed by regression analysis. Normality testing was performed beforehand using the Kolmogorov-Smirnov test, if necessary. Level of significance was set to *P*-values below 0.05. All statistical analyses were conducted on the SPSS Statistics software platform, version 26.0, by IBM Corp. (Armonk, New York, USA).

### Ethics approval and consent to participate.

We received ethical approval for this study from the ethical committee of the Antwerp University Hospital and the University of Antwerp (nr. 16/46/491). Informed consent forms signed by a parent or other legal guardian were collected from all included patients.

### Data availability.

The anonymized data sets used and/or analyzed during the current study are available from the corresponding authors on reasonable request.
